# Valorization of *Hericium erinaceus* By-Products for β-Glucan Recovery via Pulsed Electric Field-Assisted Alkaline Extraction and Prebiotic Potential Analysis

**DOI:** 10.3390/foods15010145

**Published:** 2026-01-02

**Authors:** Tannaporn Jeenpitak, Alisa Pattarapisitporn, Pipat Tangjaidee, Tabkrich Khumsap, Artit Yawootti, Suphat Phongthai, Seiji Noma, Wannaporn Klangpetch

**Affiliations:** 1 Faculty of Agro-Industry, Chiang Mai University, Chiang Mai 50100, Thailand; tannaporn.jj@gmail.com (T.J.);; 2 The United Graduate School of Agricultural Sciences, Kagoshima University, 1-21-24, Korimoto, Kagoshima 890-0065, Japan; 3 Center of Excellence in Agro Bio-Circular-Green Industry (Agro BCG), Chiang Mai University, Chiang Mai 50100, Thailand; 4 Department of Electrical Engineering, Faculty of Engineering, Rajamangala University of Technology Lanna, Chiang Mai 50300, Thailand; 5 Cluster of High Value Products from Thai Rice and Plants for Health, Chiang Mai University, Chiang Mai 50100, Thailand; 6 Lanna Rice Research Center, Chiang Mai University, Chiang Mai 50100, Thailand; 7 Faculty of Agriculture, Institute of Education and Research, College of Natural Sciences, Saga University, 1, Honjo, Saga 840-8502, Japan

**Keywords:** β-glucan, prebiotic, pulse electric field, mushroom, *Hericium erinaceus*

## Abstract

*Hericium erinaceus* is a well-known edible fungus rich in β-glucans, widely recognized for its immune-boosting and prebiotic properties. This study used a pulsed electric field (PEF) combined with alkaline extraction to improve β-glucan yield from H. erinaceus by-products. The treated residues were extracted with hot water or 7.5% NaOH. The results exhibited that PEF pretreatment followed by NaOH extraction gave the highest β-glucan yield (25 g/100 g) and purity (56.93%). SEM images revealed greater cell wall damage in NaOH-treated samples, while FTIR spectroscopy confirmed clear β-glycosidic linkages. The optimal conditions of PEF investigated by response surface methodology (RSM) were electric field strength 10 kV/cm, frequency 12 Hz, and mushroom/water ratio 8.44%, yielding β-glucan content of 50.14%. The extracted β-glucan demonstrated high prebiotic potential, supporting probiotic *Lactobacillus* spp. growth, enhancing short-chain fatty acids production, and resisting gastrointestinal digestion. Overall, this study demonstrates the broader potential of PEF-assisted alkaline extraction to support sustainable food processing, valorization of agro-industrial by-products, and the development of functional ingredients for modern food industry applications.

## 1. Introduction

*Hericium erinaceus*, commonly known as Lion’s Mane mushroom or Yamabushitake, this mushroom contains several biologically significant compounds, particularly β-glucan polysaccharides, known for their anti-cancer and immune-modulating effects [1]. However, mushrooms are highly perishable and begin to deteriorate immediately after harvest, leading to substantial postharvest losses. Moreover, mushroom by-products with low commercial value, such as misshapen, discolored, or lower-grade specimens, have become an important concern despite retaining functional and nutritional value. Nutritionally, mushrooms are a good source of protein, energy, and carbohydrates. They also contain non-starchy carbohydrates, essential minerals, vitamin B, and β-glucans, which serve as key structural components in plants and fungi [2]. β-glucans are polysaccharides found in the cell walls of higher plants and fungi, contributing to their structural integrity and biological functions [3].

Previous research confirmed the prebiotic effects of β-glucan [4,5], a non-digestible food component that promotes the growth of beneficial intestinal microorganisms and enhances host health. β-glucans improve gut health by stimulating beneficial bacteria such as Bifidobacteria and Lactobacilli. *Agaricus bisporus*, *Pleurotus ostreatus*, and *H. erinaceus* are recognized for their prebiotic properties, with pleuran and lentinan demonstrating significant efficacy in reducing intestinal inflammation and ulcers [6]. β-glucans from *Auricularia auricula-judae* significantly enhance the growth of Bifidobacteria and Lactobacilli [7]. Short-chain fatty acids (SCFAs) are the end products of anaerobic bacterial fermentation in the gastrointestinal tract. Once absorbed and metabolized, they enable the host to derive energy from food that remains undigested in the upper intestine. Under normal physiological conditions, SCFA formation depends entirely on commensal microbes, which also generate beneficial SCFAs such as acetic, butyric, and propionic acids [8]. Traditional methods for extracting bioactive compounds from mushrooms, such as hot water extraction at 95 to 100 °C, have been widely studied.

However, these techniques are often limited by high energy consumption and lengthy extraction times. Non-conventional extraction methods can improve efficiency and sustainability. One potential method is pulsed electric field (PEF) extraction, a non-thermal method that applies short, high-intensity electrical pulses to a sample placed between electrodes. These pulses create an electric charge that disrupts the cell membrane, leading to a process called electroporation, where small pores form in the membrane. As a result, the membrane loses its integrity, making it easier for important substances like polysaccharides to pass into solution [9,10,11]. PEF can significantly enhance extraction yields. Andreou et al. [12] reported an increase of up to 20.5% in tomato juice yield compared to untreated samples, while Du et al. [13] demonstrated that HIPEF-assisted extraction improved pectin yield from orange peel powder by 8.48%. Similarly, Chatzimitakos et al. [14] observed a 38% increase in blueberry juice extraction when the samples were treated with PEF at 1 or 3 kV/cm, relative to the untreated controls. Although numerous studies have reported the efficacy of PEF in enhancing the extraction of bioactive compounds, its application in extracting β-glucan from *Hericium erinaceus*, particularly in combination with alkaline extraction, remains underexplored.

Preliminary experiments showed that PEF treatment alone yielded lower β-glucan content compared to thermal or alkaline extraction methods, indicating limited efficiency when used independently. Thus, combining PEF with alkaline extraction may enhance cell wall disruption and recovery efficiency. Accordingly, this study aimed to improve polysaccharide extraction from *H. erinaceus* by-products using PEF-assisted techniques, quantify the β-glucan content, and assess the prebiotic activity of the extracts.

## 2. Materials and Methods

### 2.1. Material Preparation

By-products of *H. erinaceus* (He) harvested between September and October 2023 were generously provided by Siam Mushroom Farm, Chiang Mai, Thailand. These by-products originated from trimming residues and fruiting bodies exhibiting discoloration or non-standard shapes that were unsuitable for the current fresh market and thus classified as agricultural waste. The He by-products were dried in a hot-air oven at 45 °C for 12 h, and then powdered using a hammer mill, sieved through a 40-mesh sieve, and stored in a Ziplock bag at −18 °C.

### 2.2. Proximate Analysis

The standard proximate analysis methods described by AOAC (1995) Method were used to determine the ash (AOAC method 942.05), crude protein (AOAC method 979.09), and moisture contents (AOAC method 934.01). Crude fiber was determined using a FOSS fiber analyzer (AOAC method 978.10), and crude fat using the Soxlet extraction method (AOAC method 920.39). The carbohydrate content was calculated by Equation (1):

(1) Carbohydrate content (%) = 100−(%MC + %ash + %crude protein + %crude fiber + %crude fat)

### 2.3. Pretreatment with PEF and Extraction of Crude β-Glucan

A PEF system similar to that described by Thikham et al. [15] was used in this study. The extraction chamber consisted of parallel stainless-steel rectangular electrodes (4 × 13 cm) with 2 cm spacing. The applied electric field strength was adjusted between 5 and 10 kV/cm, with pulse frequencies ranging from 5 to 15 Hz. The PEF generator produced exponential decay pulses with a pulse duration of 0.1 microsecond and allowed voltage adjustment from 0 to 20 kV and frequency control from 0.1 to 15 Hz. The temperature increase during treatment did not exceed 30 ± 2 °C.

The He by-product powder was mixed with distilled water according to the response surface methodology (RSM) conditions, as shown in Table S1, with ratios of He/water being 5, 7.5, and 10% *w*/*w*. The mixture was treated with PEF under electric field strengths of 3, 6.5, and 10 kV/cm and frequencies of 5, 10, and 15 Hz for 14,000 pulses. The selection of 14,000 pulses was based on preliminary experiments using 10 kV/cm and 15 Hz, where different pulse numbers (12,000, 13,000, and 14,000) were compared. The 14,000 pulse condition resulted in the highest polysaccharide yield (3.43 ± 0.43%), exceeding those obtained with 12,000 (1.70 ± 0.09%) and 13,000 pulses (1.61 ± 0.10%).

The extraction conditions used in this study were based on previously published data [15]. Briefly, the minimum operating conditions of the machine are 3 kV/cm, 5 Hz, while the maximum settings are 10 kV/cm, 15 Hz. According to previous studies, the number of pulses did not significantly affect the extraction yield. Therefore, the maximum machine settings were selected for this study. After the PEF pretreatment, the supernatant (PEF-S) and precipitates (PR) were separated by centrifugation. β-glucans were recovered from the precipitates; dried in a vacuum oven at 50 °C for 12 h; and then powdered, sieved, and stored in a Ziplock bag at −18 °C. For the recovery of crude β-glucan from *H. erinaceus*, two methods were employed for comparison, which are described in the following section.

Extraction of crude β-glucan using alkaline conditions (PRA)

PR powder was mixed with 7.5% sodium hydroxide and stirred at room temperature for 24 h The extract was recovered by centrifugation at 10,956× *g*, 4 °C, for 15 min. The supernatant was collected, and the precipitate was re-extracted using the same alkali treatment [16]. The combined supernatant extracts were adjusted to pH 6 using glacial acetic acid, mixing the supernatant after the extract with the supernatant after pretreatment, and then concentrated by a rotary evaporator under the conditions of 60 °C and 80 mbar, followed by thorough dialysis against distilled water using a 10 kDa molecular weight cut-off membrane. The mixture was then mixed with 5-fold ethanol and centrifuged to recover the precipitate. Finally, the precipitate was freeze-dried.

Extraction of crude β-glucan with hot water (PRH)

Based on the method of Smiderle et al. [17], the PR powder was mixed with distilled water at a ratio of 1:30 *w*/*v* and then heated at 100 °C for 6 h The precipitates were removed by centrifugation at 10,956× *g*, 4 °C, for 15 min. The supernatant was collected and processed as described in PRA.

### 2.4. Analysis of α and β-Glucan Contents

The β-glucan content of the raw materials and the extracted β-glucan from different methods was determined using a Mushroom and Yeast Β-glucan K-YBGL kit (Megazyme, Brey, Ireland) according to the manufacturer’s instructions [18]. Briefly, the samples were milled and placed in ice-cold 12 M H_2_SO_4_ for 2 h to solubilize the glucans. Then, the samples were hydrolyzed in 2 M H_2_SO_4_ at 100 °C for 2 h After incubation, any remaining glucan fragments were quantitatively hydrolyzed to glucose using a mixture of highly purified exo-1,3-β-glucanase and β-glucosidase to give a measurement of total glucans. The α-glucan and sucrose contents of the sample were determined by specific hydrolysis to D-glucose and D-fructose. Glucose was measured with amyloglucosidase and invertase using a GOPOD reagent. The β-glucan content was calculated from the difference between the total glucan content and the α-glucan content.

### 2.5. Scanning Electron Microscopy

The residue was extracted using the PEF technique (PR), followed by hot water and alkaline extraction of the post-PEF residue (PRH, PRA). All the samples were then desiccated in a hot-air oven at 45 °C for 24 h Scanning electron microscopy (SEM) specimen holders with samples were coated with Pd/Pt (palladium-platinum) three times. The analyses were carried out at an accelerating voltage of 5 kV, with micrographs taken at 500×, and 2000× to examine the surface properties using an S-3400N SEM (Hitachi, Ibaraki, Japan).

### 2.6. Fourier-Transform Infrared Spectroscopy

Fourier-transform infrared (FTIR) spectroscopy was performed using a Bruker FTIR Vertex 70 v instrument (Billerica, MA, USA) equipped with attenuated total reflectance (ATR). The samples were placed on the ATR crystal. Measurements were taken within the wavenumber range of 500–4000 cm^−1^ to achieve ultra-high vacuum conditions at a resolution of 20 cm^−1^.

### 2.7. Evaluation of Prebiotic Potential

#### 2.7.1. Analysis of Lactic Acid Bacterial Growth Promotion

The method used to assess the ability to promote the growth of target lactic acid bacteria (LAB) was adapted from Thikham et al. [15]. The target LAB strains used in this study were carefully selected based on prior screening. These included *Levilactobacillus brevis* TISTR 860 from the Thailand Institute of Science and Technology Research; *Lactobacillus bugaricus* JCM 1002, *L. sakeii* JCM 1157, *L. crispatus* JCM8780, and *Lactiplantibacillus plantarum* JCM 1149T from the Japan Collection of Microorganisms; and *Limosilactobacillus* (*Lactobacillus*) *reuteri* KUB-AC5 and *Lactococcus lactis* KA-FF 1–4 from Dr. Massalin Nakphaichit.

A starter culture was prepared by incubating the bacteria in MRS broth (Difco, Las Vegas, NV, USA) at 37 °C for 18 h. The bacterial inoculum was then adjusted to 3 Log CFU/mL in 0.85% normal saline and added to the modified MRS (mMRS). In the mMRS broth, the original carbon source was replaced with β-glucan from *H. erinaceus* by-products (BG-He) to achieve a final concentration of 2% (*w*/*v*). The mMRS comprised a carbon source 2 g/L, peptone 10 g/L, polysorbate 80 (Tween 80) 1 mL/L, ammonium citrate 2 g/L, CH_3_COONa 5 g/L, MgSO_4_ 0.1 g/L, MnSO_4_ 0.05 g/L, and K_2_HPO_4_ 2 g/L. The culture was incubated at 37 °C for 24 h The bacterial count was analyzed using the single plate-serial dilution spotting (SP-SDS) technique and compared with the mMRS without a carbon source (as a negative control) and the mMRS with commercial β-glucan (BGC), inulin, and glucose (as a positive control).

#### 2.7.2. Short-Chain Fatty Acids (SCFAs) Content

The SCFAs were analyzed based on the method described by De Baere et al. [19]. The selected LAB were incubated for 0 and 16 h and centrifuged at 15,493× *g* for 15 min at 4 °C. The clearer supernatants obtained were then diluted with HPLC-grade water in a 1:1 ratio. Lactic acid and SCFAs were measured using high-performance liquid chromatography (HPLC) with an Aminex HPX 87H column (Bio-Rad, Hercules, CA, USA), 5 mM H_2_SO_4_ as the mobile phase, a flow rate of 0.75 mL/min, and a UV detector set at 210 nm. The concentrations of lactic acid and SCFAs were determined by comparing the peak areas to standard solutions of lactic, acetic, propionic, and *n*-butyric acid.

#### 2.7.3. In Vitro Resistance to Gastric and Small Intestinal Digestion

The digestion properties were evaluated using in vitro gastric acid and simulated gastric juice, following the modified method proposed by Minekus et al. [20] using 2% BG-He in simulated gastric fluid buffer (SGF) comprising KCl 6.9 mM, KH_2_PO_4_ 0.9 mM, NaHCO_3_ 25 mM, NaCl 47.2 mM, MgCl_2_ (H_2_O) 6.01 mM, and CaCl_2_(H_2_O)_2_ 0.15 mM. Then, 7.5 mL SGF, pepsin 25,000 U/mL (1.6 mL), 0.3 M CaCl_2_ (5 μL), and 1 M HCl (0.2 mL) were added to 10 mL of BG-He solution to adjust the pH to 2.0, followed by the addition of 0.695 mL of water. The sample mixture was incubated at 37 °C for 2 h. Subsequently, 20 mL of the sample mixture was combined with 11 mL of simulated intestinal fluid (SIF) containing KCl 6.8 mm, KH_2_PO_4_ 0.8 mm, NaHCO_3_ 85 mg, NaCl 38.4 mm, MgCl_2_(H_2_O)_6_ 0.33 mm, and CaCl_2_(H_2_O)_2_ 0.6 mm. To this mixture, pancreatin 800 U/mL (0.5 mL), 160 mM bile salt (2.5 mL), 0.3 M CaCl_2_ (40 μL), and 1 M NaOH (0.15 mL) were added to adjust the pH to 6.9, followed by the addition of 1.31 mL of water. The sample mixture was incubated at 37 °C for 2 h. At 2 and 4 h, 1 mL of the sample was collected and analyzed for reducing sugar content using the DNS method [21] and total sugar by the phenol–sulfuric acid method [22]. The hydrolysis percentage was calculated by comparing with BGC and inulin as a control group using Equation (2):

(2) $$\mathrm{Hydrolysis}\;(\%)\;=\;\frac{\mathrm{final}\;\mathrm{reducing}\;\mathrm{sugar}\;-\mathrm{initial}\;\mathrm{reducing}\;\mathrm{sugar}}{\mathrm{total}\;\mathrm{sugar}\;-\;\mathrm{initial}\;\mathrm{reducing}\;\mathrm{sugar}}\;\times \;100$$

### 2.8. Statistical Analysis

Statistical analysis was conducted using the SPSS version 17.0 software (SPSS Inc., Chicago, IL, USA). Mean values were compared using Duncan’s multiple range test at a 95% confidence level, with the results expressed as the mean of three replicates ± standard deviation.

## 3. Results and Discussion

### 3.1. Chemical Composition

The proximate composition of He powders, which were oven-dried at 45 °C for 6 h and milled to a fine powder prior to analysis, is shown in Table S2 as moisture content 4.90, ash 4.45, protein 7.42, fat 1.7, and fiber 15.68 g per 100 g sample (wet basis). The fiber content observed in this study concurred with Gonkhom et al. [23], who reported fiber content in four mushroom strains ranging from 10.96 to 11.68%DW. The carbohydrate content was 65.79%, with β-glucan content in the raw material 43.54% *w*/*w*, consistent with the findings of Chen et al. [24], who reported β-glucan content of 68% *w*/*w* in *H. erinaceus*.

### 3.2. Effect of PEF, Hot Water, and Alkali on β-Glucan Extraction from H. erinaceus Mushroom By-Products

The preliminary results on yield percentage and β-glucan content in the extracts from different methods after pretreatment of He are shown in Table 1. The yield in the PEF-S was 3.43 g/100 g dried He; however, PEF-assisted extraction with PRH and PRA extraction yielded 4.73 and 25.00 g/100 g dried He, respectively, and the hot water extraction was 3.59 g/100 g. The samples treated with PRA and PRH gave β-glucan contents of 56.93 and 68.05% *w*/*w*, respectively. Khoshyaran et al. [25] reported that alkali extraction of β-glucan from baker’s yeast using 0.6 M NaOH, at 70 °C, with stirring at 100 rpm for 7 h, resulted in β-glucan purity of 85% *w*/*w*. Conventional methods, such as hot water extraction, typically resulted in lower yields. Sakdasri et al. [26] reported that β-glucan extracted from *Ganoderma lucidum* using pressurized hot water at 200 °C, 100 bar for 180 min yielded β-glucan at only 0.44 mg/100 g dried sample and total polysaccharide 5.36 g/100 g. Yoo et al. [27] found that increasing the extraction time during pressurized hot water treatment degraded β-glucan. Although PEF facilitated cell wall disruption through electroporation and both PRH and PRA supported efficient β-glucan recovery, hot water extraction still resulted in lower yields and required high temperature and pressure to achieve sufficient cell disruption even after PEF pretreatment. PRA, despite yielding a slightly lower β-glucan percentage compared to PRH, provided a markedly higher extraction yield, resulting in an overall product quantity that was 5.28-fold greater. Therefore, PRA was selected as the optimal extraction following PEF pretreatment to evaluate differences in β-glucan content using the Box–Behnken Design model, and the Box-Behnken design matrix factors for PEF pretratmeant are shown in Table 2.

Recent advances in β-glucan extraction technologies, such as enzymatic hydrolysis and microwave-assisted extraction, have demonstrated improved polysaccharide release through selective cell wall degradation and enhanced energy transfer. Compared with these techniques, PEF-assisted alkaline extraction offers a distinct advantage by inducing electroporation-mediated permeability at electric field strengths ranging from 1 to 10 kV/cm. This exposure promotes pore formation in the cell membrane, thereby increasing membrane permeability and facilitating the release of intracellular components. As a non-thermal process [28,29], PEF, when combined with chemical solubilization, enables efficient β-glucan extraction under lower thermal intensities. Although enzymatic extraction generally provides high β-glucan purity, it is often associated with higher costs and longer processing times [30]. Microwave-assisted extraction, while offering rapid processing, may result in partial degradation of heat-sensitive polysaccharides and typically requires high capital investment in specialized equipment, which can limit large-scale industrial application [31]. Therefore, PEF-assisted alkaline extraction represents a promising balance between extraction efficiency, cost-effectiveness, and preservation of functional properties.

### 3.3. Microstructural Changes

**Figure 1:**
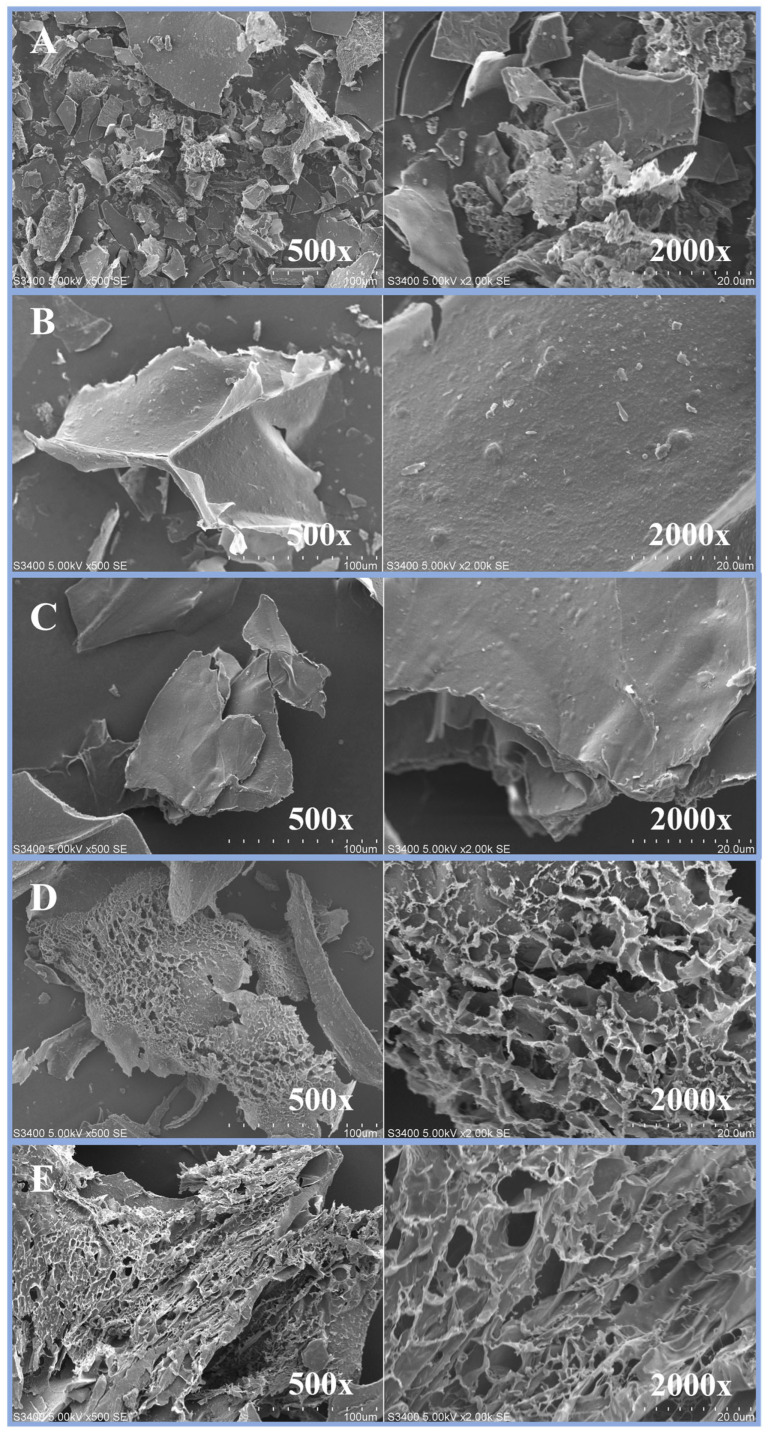
SEM micrograph of *H. erinaceus* extraction using various techniques. The images show cell structural characteristics: PR (**A**), He + H (**B**), He + A (**C**), PRH (**D**), and PRA (**E**) at magnifications of 500× and 2000×.

Scanning electron microscopy (SEM) was employed to observe the surface morphology of the samples at magnifications ranging from 500× and 2000×, providing essential insights into the extent of physical and structural damage caused by PEF, hot extraction, and alkali extraction. Morphological changes were closely associated with the disruption of cellular integrity, which influenced the efficiency of the extraction processes. The SEM images are presented in Figure 1. In Figure 1A,B, the PR sample exhibited sheet-like surface structures with scattered fragments at magnifications of 500× and 2000×, reflecting the rigid and intact nature of the fungal cell wall matrix. In mushrooms, this matrix is primarily composed of chitin, β-glucans, and glycoproteins [32], which limits solvent penetration and β-glucan release. PEF treatment enhances extraction efficiency by inducing electroporation, increasing membrane permeability, and weakening the fungal cell wall structure. Alterations in the sheet structures suggested that the PEF process induced electroporation that enhanced cell membrane permeability by generating numerous pores, thereby facilitating the release and subsequent extraction of β-glucan.

By contrast, in the He power extract by hot water extraction (He + H, B) and the He power extract by alkaline extraction (He + A, C) samples, the cell wall surfaces at high magnifications remained smooth and non-porous. Carullo et al. [33] reported that PEF treatment caused the microalgal cell structure to lose its shape when exposed to applied energy, allowing the cells to release intracellular compounds through the porous cell membrane. Calleja-Gómez et al. [34] reported that treating *Agaricus bisporus* mushrooms with PEF and conventional water extraction caused more damage to the microstructure compared with hot water extraction.

Significant surface changes were observed when the PEF residues were treated with hot water and alkali. The PRH samples (D), viewed at magnifications between 500× and 2000×, displayed a sheet-like structure with small, broken fragments. Porosity was only evident in certain areas, and the sample retained a strong structure with minimal internal fiber breakage (D). By contrast, the PRA samples (E) exhibited more extensive damage to the cell wall, with clear penetrations and visible porosity throughout, indicating breakdown of the internal cell structure, which facilitated extraction. This structural alteration was consistent with Putranto et al. [35], who reported that using PEF in combination with NaOH increased the cellulose content in corn cobs. An electric field strength of 9 kV/cm effectively enhanced the cellulose content. Additionally, Calleja-Gomez et al. [34] reported that PEF pretreatment may induce greater pore formation in mushroom cell structures than heat treatment. In agreement with this observation, SEM images in the present study showed micropores on the surface of the PEF-treated samples, suggesting increased cell wall permeability. At the microstructural level, electroporation is considered to enhance solvent penetration into intracellular regions, thereby facilitating compound recovery. The combined PEF and alkaline treatment further intensified structural disruption, supporting improved β-glucan extraction.

### 3.4. Fourier-Transform Infrared Spectroscopy for Glucan Characterization

**Figure 2:**
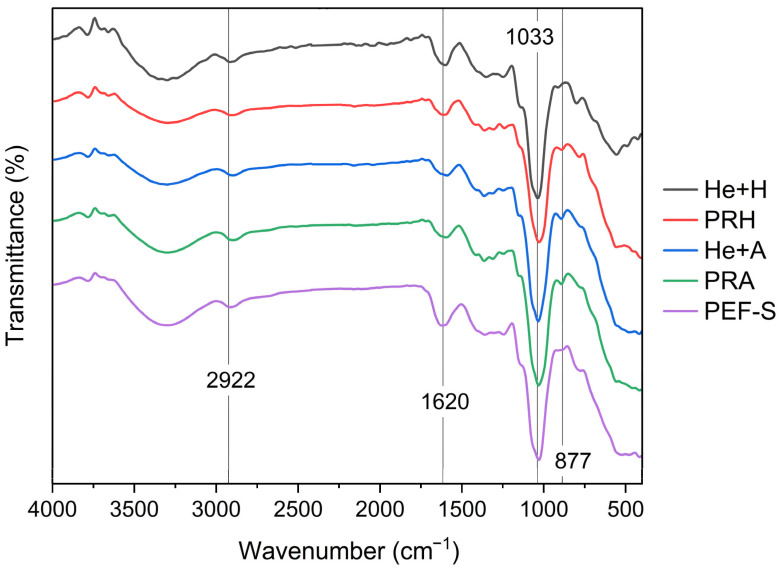
FTIR spectra of β-glucan extracted from *H. erinaceus* by He + H: *H. erinaceus*, PRH, He + A, PRA, and PEF-S.

The FTIR spectra of β-glucan extracted from *H. erinaceus* using PR, PRA, He + A, PRH, and He + H are shown in Figure 2. FTIR spectroscopy is an effective technique for observing structural modifications in biopolymers [36]. The same FTIR absorption bands were found in all the samples. The C–H and CH_2_OH stretching bands at 2922 cm^−1^, assigned to the pyranoid rings, exhibited no change. The band at 1620 cm^−1^, indicating the presence of chitin or acetamide, was consistent with Šandula et al. [36]. The samples treated with PRA, He + A, and PRH showed a new band at 887 cm^−1^, associated with β-D anomers. This peak was attributed to C–H deformation and ring vibrations, typical of β-glycosidic linkages in polysaccharides. β-Glycosidic linkages in glucans appeared at 865–837 cm^−1^ [37,38]. The β-anomer refers to a configuration where the anomeric hydroxyl group is positioned on the opposite side of the ring from the reference hydroxyl group, influencing FTIR absorption patterns [39]. PRA showed more distinct β-glycosidic bands, suggesting more effective exposure of the active structure of β-glucan.

### 3.5. Optimization of Extraction Conditions Using Response Surface Methodology (RSM)

An RSM experimental design was used to assess the effects of three independent variables, electric field strength (A), frequency (B), and the mushroom/water ratio (C) on β-glucan content, with the results shown in Table 3. The regression model obtained from the experimental data was expressed as

Y = 50.9 + 3.65A + 0.227B − 0.233C + 0.326A^2^ − 2.54B^2^ − 3.31C^2^ − 0.145AB + 1.77AC + 1.23BC

ANOVA analysis showed that the quadratic model was statistically significant (F = 4.75, *p* = 0.0261), confirming that it provided a good fit for the experimental data. The coefficient of determination (R^2^ = 0.859) further supported the adequacy of the model for predicting β-glucan content based on the selected process variables. Electric field strength (A) exhibited a strong linear effect (*p* = 0.00214), and the quadratic terms of frequency (B^2^) and the ratio (C^2^) were also significant (*p* = 0.0483 and 0.0169, respectively). Other model terms were not significant (*p* > 0.05). The lack of fit was not significant (*p* = 0.217), indicating that the model reliably described the response behavior under the tested conditions.

**Figure 3:**
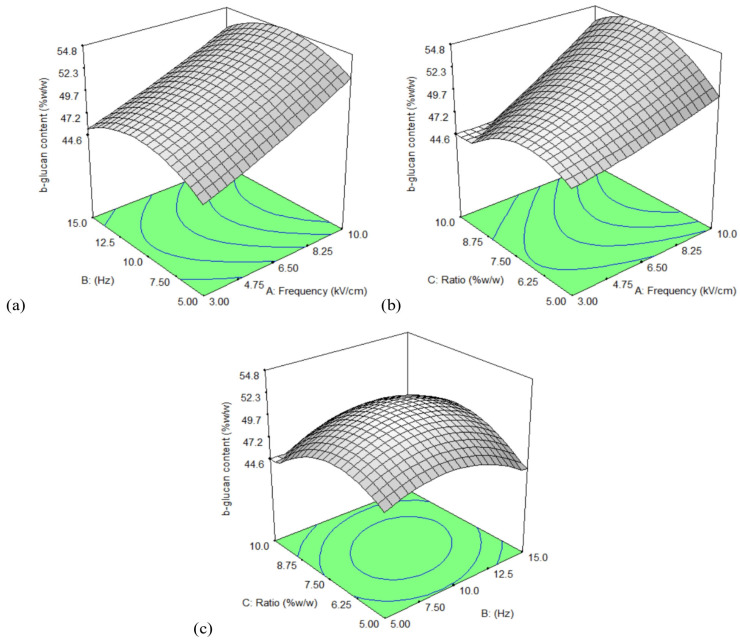
Response surface plots of β-glucan interaction between the three treatment parameters: (**a**) frequency (Hz) and electric field strength (kV/cm), (**b**) ratio and electric field strength (kV/cm), and (**c**) ratio and frequency (Hz).

The response surface plots (Figure 3) further illustrate these interactions, showing that β-glucan content increased markedly with higher electric field strength across all parameter combinations. The interaction between field strength and ratio also displayed a clear upward trend, with moderate ratios providing the most favorable conditions for mass transfer. Although frequency had a comparatively smaller effect, β-glucan yield still increased slightly at higher frequencies when combined with appropriate field strengths or ratios. Overall, the three plots confirm that electric field strength plays the dominant role in enhancing β-glucan recovery, supported by optimal combinations of frequency and extraction ratio. The optimal conditions for PEF pretreatment and alkali extraction (PRA) were electric field strength 10 kV/cm, frequency 12 Hz, and mushroom/water ratio 8.44%. Under these conditions, the β-glucan content was 50.14%, with a yield of 27.13%. The relative errors between the predicted and actual values for β-glucan under these optimized conditions are shown in Table S1. Due to limitations in equipment precision, the actual parameters were adjusted to electric field strength of 10 kV/cm, frequency of 12 Hz, and mushroom/water ratio of 8.44%, resulting in a relative error of 8.51%.

### 3.6. Lactic Acid Bacterial Growth Promotion

**Figure 4:**
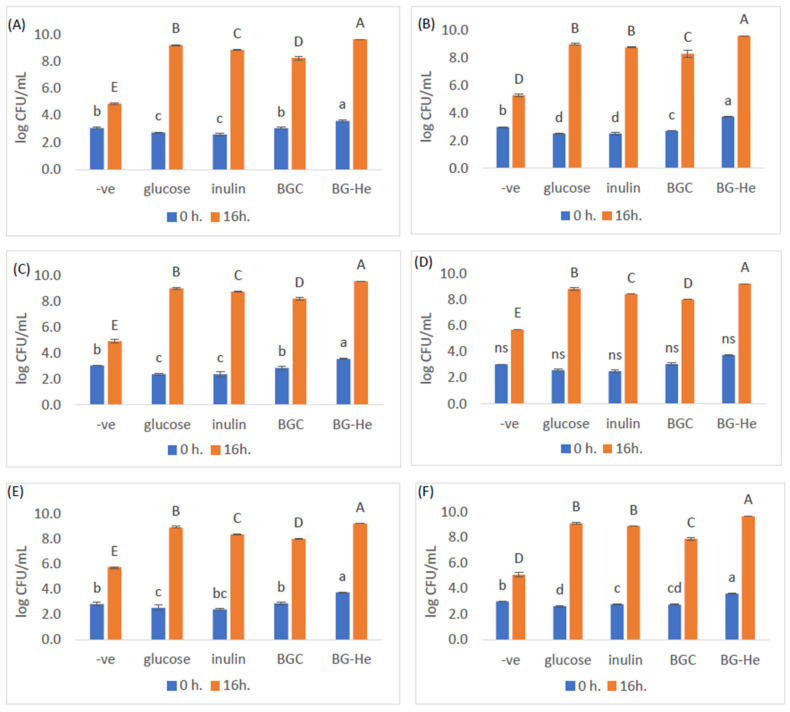
Growth enhancement of LAB cultured in mMRS medium with various carbon sources at 37 °C for 0 and 16 h, analyzed using the SP-SDS technique. BG-He: β-glucan from *H. erinaceus*, BGC: β-glucan from yeast, and -ve: negative control/no carbon source. Increase in growth of LAB incubated in (**A**) *Lactococcus lactis* KAFF-14, (**B**) *Limosilactobacillus reuteri* KUB-AC5, (**C**) *Lactobacillus crispatus* JCM8780, (**D**) *Lactobacillus plantarum* JCM1149T, (**E**) *Lactobacillus sakei* JCM1157, and (**F**) *Lactobacillus brevis* TISTR860. Note: Values are expressed as mean ± standard deviation from triplicate experiments. The lowercase letters a–d indicate significant differences at 0 h of incubation, while the uppercase letters A–E indicate significant differences at 16 h (*p* < 0.05). ns: not significant.

Figure 4 shows the growth enhancement of the six LAB (*L. lactis*, *L. reuteri*, *L. crispatus*, *L. plantarum*, *L. sakei*, and *L. brevis*) cultured in mMRS medium with glucose, inulin, BGC, and BG-He carbon sources and incubated at 37 °C for 0 and 16 h After 16 h of incubation, all six bacterial strains showed increased growth, with BG-He significantly the highest (*p* < 0.05), followed by BGC and inulin. Glucose, despite being a fermentable sugar, showed only moderate growth, while the negative control (-ve) gave the lowest bacterial promotion. BG-He showed a significant ability to promote microbial growth. These findings were consistent with Thikham et al. [15], who reported that β-glucan extracted from mushrooms using the PEF technique promoted the growth of *L. lactis*, *L. plantarum*, *L. sakei*, and *L. brevis*. Russo et al. [40] showed that a specific type of β-glucan (2-substituted (1,3)-β-D-glucan) produced by *Pediococcus parvulus* 2.6 acted as a prebiotic and improved the growth and survival of probiotic bacteria, being effective for *Lactobacillus plantarum* commonly used in functional foods, while Zhang et al. [41] found that β-glucan acted as a prebiotic to support the growth of broiler chickens.

**Figure 5:**
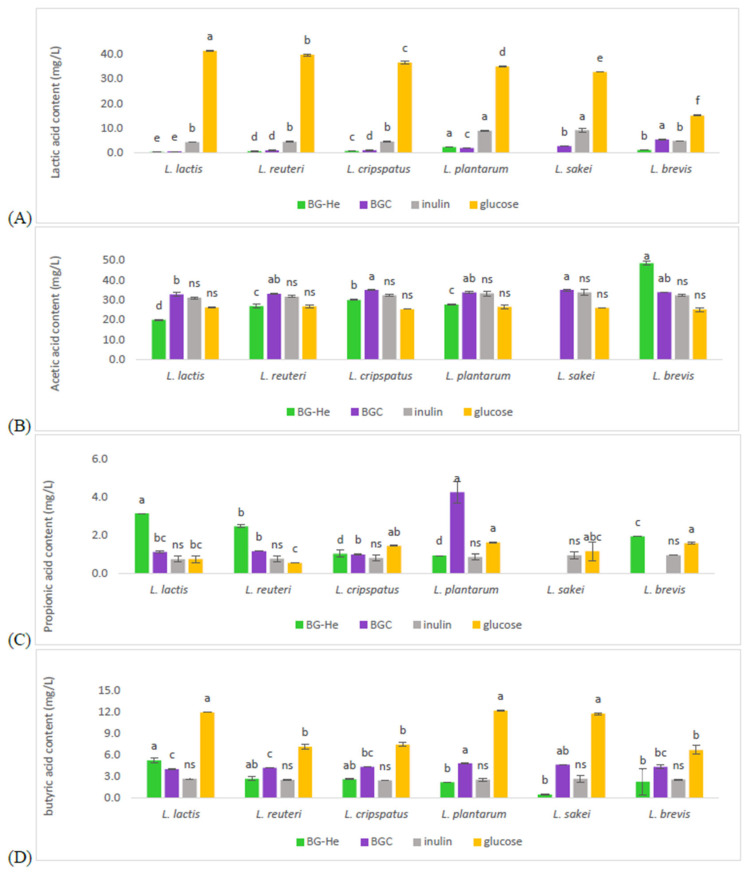
Lactic acid and SCFA production by LAB cultured with different carbon sources assessed after incubation at 37 °C for 16 h The analysis was performed using an HPLC equipped with an Aminex HPX-87H column, with 5 mM H_2_SO_4_ as the mobile phase, a flow rate of 0.75 mL/min, and a UV detector set at 210 nm. (**A**) lactic acid, (**B**) acetic acid, (**C**) propionic acid, and (**D**) butyric acid. Note: Data are expressed as mean ± standard deviation. Different lowercase letters (a–f) indicate significant differences in SCFAs production among the bacterial strains under the same carbon source (*p* < 0.05). ns: not significant.

After fermentation for 16 h, the SCFA content was analyzed using HPLC. *L. brevis* grew with BG-He as the carbon source and produced more acetic acid than the other tested strains and glucose, inulin, and BGC carbon sources. This suggested that BG-He was the most suitable to support acetic acid production (Figure 5B). mMRS supplemented with BG-He significantly increased propionic acid production by *L. lactis* and *L. reuteri*, as shown in Figure 5C compared to glucose, inulin, and BGC. *L. brevis* also produced a significantly higher amount of propionic acid in the BG-He medium. *L. lactis* cultured with BG-He showed higher production of butyric acid (Figure 5D) than that cultured with BGC. However, the amount produced was still significantly lower than in the glucose medium. Overall, BG-He promoted SCFA production more effectively than inulin and BGC in most strains, especially for acetic and propionic acids. Our results concurred with Hong et al. [42], who reported on the effects of oral β-glucan supplementation in rats. They found that rats receiving β-glucan had higher levels of acetate, propionate, and butyrate compared to the control group, suggesting that β-glucan stimulated the growth of LAB in the colon. β-Glucan was fermented by gut microbiota to generate acetate, propionate, and butyrate, while the proportion of Lactobacillus in the gut significantly increased [43]. BG-He is easier for some strains to ferment and contains components that support SCFA biosynthesis. Butyric acid was not the highest acid in BG-He but the increased production of acetic and propionic acids is important because these acids maintain gut health by lowering pH, reducing harmful bacteria, and providing energy to colon cells [44,45]. BG-He showed potential as a prebiotic carbon source for functional foods by enhancing probiotic activity and supporting intestinal health.

### 3.7. In Vitro Gastrointestinal Resistance of BG-He

Table 4 presents the resistance of BG-He to simulated gastrointestinal digestion compared with BGC and inulin. During the gastric phase, BG-He underwent more extensive hydrolysis than inulin, indicating lower resistance in the stomach. However, in the small intestinal phase, BG-He maintained a substantial undigested fraction, showing digestion behavior in the intestine that still allows it to reach the lower gut. Several studies have reported that β-glucans resist digestion in the upper gastrointestinal tract, allowing them to reach the small intestine and interact with the mucus layer [46]. Mackie et al. [47] reported that 90% of oat bran β-glucan was released in the proximal small intestine during in vitro digestion, suggesting that it is mostly undigested in the stomach. This resistance enables β-glucans to support gut barrier function and act as prebiotics by serving as substrates for beneficial bacteria in the lower intestine. This resistance is an important property of prebiotic fibers because it allows them to act as substrates for beneficial bacteria in the large intestine. The digestion of BG-He indicates that, despite partial gastric hydrolysis, it can reach the colon to be fermented by LAB, producing SCFAs that promote gut health and exhibit prebiotic potential. Therefore, the partial resistance of BG-He to gastrointestinal digestion may contribute to its prebiotic function and support its application in functional food products.

## 4. Conclusions

This study investigated the chemical compositions and β-glucan extraction methods from *H. erinaceus* mushroom by-products. PRA gave high β-glucan content and extraction yield. This method also caused the most severe damage to mushroom cell walls, releasing more β-glucan. The FTIR analysis confirmed that all the extraction methods produced similar chemical structures, with β-glycosidic bands more noticeable in PRA. In the optimization RSM study, electric field strength and frequency had a stronger impact on β-glucan yield than the ratio of He to water. Model predictions under the optimized conditions of electric field strength 10 kV/cm, frequency 12 Hz, and ratio 8.44% closely matched the experimental results, with a relative error of 8.51% for β-glucan yield. BG-He significantly supported the growth of LAB and improved the production of SCFAs, especially acetic and propionic acids, which are beneficial for gut health. The hydrolysis test results indicated that BG-He retained a significant undigested fraction through the upper gastrointestinal tract, supporting its potential as a prebiotic ingredient. Therefore, BG-He shows promise as a natural prebiotic that can support the growth of probiotic bacteria and improve gut health.

## Figures and Tables

**Table 1:** Preliminary results of yield and β-glucan content from heat and alkali extraction of *H. erinaceus*.

Sample	Yield (g/100 g)	β-Glucan Content (% *w*/*w*)
He		43.54 ± 0.64 ^c^
PEF-S	3.43 ± 0.43 ^b^	25.73 ± 4.63 ^d^
PRH	4.73 ± 1.38 ^b^	68.05 ± 4.24 ^a^
PRA	25.00 ± 2.96 ^a^	56.93 ± 5.07 ^b^

Note: Values are expressed as mean ± standard deviation from triplicate experiments. Different lowercase letters (a–d) within each column indicate significant differences (*p* < 0.05). He, *H. erinaceus* powder; PEF-S, supernatant of *H. erinaceus* after PEF pretreatment; PRH, PEF residues extracted with hot water; PRA, PEF residues extracted with 7.5% NaOH.

**Table 2:** Box-Behnken design matrix factors for the pretreatment of HE using the PEF technique.

Factor	Symbol	Levels		
		Low (−1)	Intermediate (0)	High (+1)
Electric field strength (kV/cm)	A	3	6.5	10
Frequency (Hz)	B	5	10	15
Mushroom/water ratio (% *w*/*w*)	C	5	7.5	10

**Table 3:** Relative errors between the predicted and actual values for β-glucan extraction under optimized PEF and alkali conditions.

Value	Optimized Process Parameters			Response
	Electric Field Strength (kV/cm)	Frequency (Hz)	Ratio (% *w*/*v*)	β-Glucan (% *w*/*w*)
Predicted	9.99	12.2	8.44	54.80
Actual	10	12	8.44	50.14 ± 1.36
Error (%)	0.10	1.64	0.00	8.51

Note: Error (%) = ((Predicted − Actual)/Predicted) × 100. Values are expressed as mean ± standard deviation from triplicate experiments.

**Table 4:** Hydrolysis percentage of BG-He in simulated gastric phase and small intestinal phase conditions compared with BGC and inulin.

Sample	Hydrolysis (%)	
	Gastric Phase	Small Intestine Phase
BG-He	9.26 ± 0.79 ^a^	16.55 ± 1.11 ^a^
BGC	1.82 ± 0.31 ^c^	11.01 ± 2.13 ^b^
Inulin	6.88 ± 0.29 ^b^	16.89 ± 0.60 ^a^

Note: Values are expressed as mean ± standard deviation from triplicate experiments. Different superscripts a, b, and c indicate significantly different values (*p* < 0.05). BG-He: β-glucan from *H. erinaceus*, BGC: commercial β-glucan from yeast.

## Data Availability

The original contributions presented in this study are included in the article/Supplementary Materials. Further inquiries can be directed to the corresponding author.

## Supplementary Materials

The following supporting information can be downloaded at https://www.mdpi.com/article/10.3390/foods15010145/s1. Table S1: Three-factor Box–Behnken Design matrix with observed and predicted β-glucan (% *w*/*w*) after PEF pretreatment and alkali extraction; Table S2: Chemical composition of *Hericium erinaceus*.
