# Supplementary material for: Valorization of Hericium erinaceus By-Products for β-Glucan Recovery via Pulsed Electric Field-Assisted Alkaline Extraction and Prebiotic Potential Analysis
Source: Foods. 2026 Jan 2;15(1):145. doi: 10.3390/foods15010145 (PMC12785338; doi:10.3390/foods15010145)
Supplement: Supplementary file 1 [file foods-15-00145-s001.zip › foods-4043951-supplementary.pdf]

Table S1. Three-factor Box–Behnken Design matrix with observed and predicted β-glucan (% *w*/*w*) after PEF pretreatment and alkali extraction.

Run	Factors			Response β-glucan (% *w/w*)	
	A	B	C	Observed [a]	Predicted
1	0	0	0	47.89±1.26	50.9
2	1	0	-1	50.81±1.49	50.0
3	-1	-1	0	46.89±1.02	44.7
4	0	0	0	51.07±1.01	50.9
5	0	1	1	47.06±0.39	46.3
6	1	1	0	50.12±0.16	52.4
7	0	1	-1	45.67±0.56	44.3
8	0	0	0	51.33±0.97	50.9
9	-1	1	0	45.38±1.03	45.4
10	1	0	1	54.50±0.83	53.1
11	0	0	0	51.59±1.01	50.9
12	1	-1	0	52.21±1.93	52.3
13	-1	0	-1	44.80±0.64	46.3
14	0	-1	-1	45.42±1.47	46.3
15	-1	0	1	41.39±1.83	42.3
16	0	-1	1	41.89±1.75	43.4
17	0	0	0	52.45±1.39	50.9

Note: [a] All the treatments were performed in 3 replication, and the observed results were reported as mean ± standard deviation from the experiment. A:Electric fields strength, B: Frequency, C: Ratio.

Table S2. Chemical composition of *Hericium erinaceus*

**Chemical composition**	**He (g/100g sample)**
Ash	4.45 ± 0.13[e]
Moisture content	4.90 ± 0.08[d]
Fiber	15.68 ± 0.37[b]
Fat	1.78 ± 0.07[f]
Protein	7.42 ± 0.06[c]
Carbohydrate	65.79 ± 0.28[a]

Note: Data values are expressed as mean ± standard deviation from the experiment. Difference letters a-f indicate significantly different values ($P < 0.05$).
